# A Machine Learning Method for Allocating Scarce COVID-19 Monoclonal Antibodies

**DOI:** 10.1001/jamahealthforum.2024.2884

**Published:** 2024-09-13

**Authors:** Mengli Xiao, Kyle C. Molina, Neil R. Aggarwal, Laurel E. Beaty, Tellen D. Bennett, Nichole E. Carlson, Lindsey E. Fish, Mika K. Hamer, Bethany M. Kwan, David A. Mayer, Jennifer L. Peers, Matthew K. Wynia, Adit A. Ginde

**Affiliations:** 1 Department of Biostatistics and Informatics, Colorado School of Public Health, University of Colorado Anschutz Medical Campus, Aurora; 2 Department of Emergency Medicine, University of Colorado School of Medicine, Aurora; 3 Division of Pulmonary Sciences, Department of Medicine, University of Colorado School of Medicine, Aurora; 4 Department of Informatics and Data Science, University of Colorado School of Medicine, Aurora; 5 Colorado Clinical and Translational Sciences Institute, University of Colorado Anschutz Medical Campus, Aurora; 6 Department of Pediatrics, University of Colorado School of Medicine; University of Colorado Anschutz Medical Campus, Aurora; 7 Division of General Internal Medicine, Denver Health, Denver, Colorado; 8 Division of General Internal Medicine, University of Colorado School of Medicine, Aurora; 9 Center for Bioethics and Humanities, University of Colorado Anschutz Medical Campus, Aurora; 10 Department of Family Medicine, University of Colorado School of Medicine, Aurora

## Abstract

**Importance:**

During the COVID-19 pandemic, the effective distribution of limited treatments became a crucial policy goal. Yet, limited research exists using electronic health record data and machine learning techniques, such as policy learning trees (PLTs), to optimize the distribution of scarce therapeutics.

**Objective:**

To evaluate whether a machine learning PLT-based method of scarce resource allocation optimizes the treatment benefit of COVID-19 neutralizing monoclonal antibodies (mAbs) during periods of resource constraint.

**Design, Setting, and Participants:**

This retrospective cohort study used electronic health record data from October 1, 2021, to December 11, 2021, for the training cohort and data from June 1, 2021, to October 1, 2021, for the testing cohort. The cohorts included patients who had positive test results for SARS-CoV-2 and qualified for COVID-19 mAb therapy based on the US Food and Drug Administration’s emergency use authorization criteria, ascertained from the patient electronic health record. Only some of the qualifying candidates received treatment with mAbs. Data were analyzed between from January 2023 to May 2024.

**Main Outcomes and Measures:**

The primary outcome was overall expected hospitalization, assessed as the potential reduction in overall expected hospitalization if the PLT-based allocation system was used. This was compared to observed allocation using risk differences.

**Results:**

Among 9542 eligible patients in the training cohort (5418 female [56.8%]; age distribution: 18-44 years, 4151 [43.5%]; 45-64 years, 3146 [33.0%]; and ≥65 years, 2245 [23.5%]), a total of 3862 (40.5%) received mAbs. Among 6248 eligible patients in the testing cohort (3416 female [54.7%]; age distribution: 18-44 years, 2827 [45.2%]; 45-64 years, 1927 [30.8%]; and ≥65 years, 1494 [23.9%]), a total of 1329 (21.3%) received mAbs. Treatment allocation using the trained PLT model led to an estimated 1.6% reduction (95% CI, −2.0% to −1.2%) in overall expected hospitalization compared to observed treatment allocation in the testing cohort. The visual assessment showed that the PLT-based point system had a larger reduction in 28-day hospitalization compared with the Monoclonal Antibody Screening Score (maximum overall hospitalization difference, −1.0% [95% CI, −1.3% to −0.7%]) in the testing cohort.

**Conclusions and Relevance:**

This retrospective cohort study proposes and tests a PLT method, which can be linked to a electronic health record data platform to improve real-time allocation of scarce treatments. Use of this PLT-based allocation method would have likely resulted in fewer hospitalizations across a population than were observed in usual care, with greater expected reductions than a commonly used point system.

## Key Points

**Question:**

Can the use of a policy learning–based allocation method improve population health benefits achieved when allocating scarce treatments?

**Findings:**

This retrospective cohort study examined methods for allocating scarce neutralizing monoclonal antibodies, using electronic health record data from more than 15 000 patients with COVID-19 within a large health care system, and found that a policy learning tree–based allocation method would potentially have resulted in lower hospitalization rates compared to the observed data. Further, a point system based on the policy learning trees outperformed another commonly used point-scoring system.

**Meaning:**

Using electronic health record data to show that machine learning methods, namely policy learning trees, can improve the allocation of scarce therapeutics; therefore, policy learning tree–based allocation should be considered in potential future episodes of therapeutic scarcity, including pandemics.

## Teaser

This retrospective cohort study evaluates a machine learning–based policy learning tree method for allocating scarce resources to maximize the treatment benefits of COVID-19 neutralizing monoclonal antibodies during times of resource constraints.

## Introduction

The COVID-19 pandemic placed an unprecedented burden on modern health care systems, leading to shortages in critical medications, hospital beds, masks, ventilators, workforce, and many other resources. As scarcities arose, policymakers faced challenging decisions regarding the allocation of limited resources. Especially early in the pandemic, allocation policies were made with remarkably incomplete information, as little was known about COVID-19 transmission, disease course, and emerging therapeutic effectiveness. Still, policymakers sought to optimize key ethical values of equity and efficiency in developing allocation protocols, seeking to save the most lives while alleviating, or at least not exacerbating, health disparities.^1^

Neutralizing monoclonal antibodies (mAbs) emerged as the initial outpatient therapeutic that could reduce the progression of mild to moderate COVID-19 to severe disease and hospitalization. During peak pandemic periods, mAb administration was constrained by scarcity not only of mAbs, but also of dedicated personnel for intravenous infusions, segregated space, and other key resources, which led to rationing of mAb treatment across the US. Evidence-based mAb allocation policies were hampered by limited mAb-specific data, and the criteria used for US Food and Drug Administration (FDA) emergency use authorization (EUA) were far broader than the populations studied in clinical trials. Accordingly, health care systems and national guidance used expert consensus or, if local data were available, simple regression-based tools to aid in allocation policies.^2^ However, these approaches may fail to account for the complex interactions that may influence progression to severe disease and hospitalization.

Policy learning (PL) is a machine learning method that can leverage observational data to find treatment allocation rules that optimize a population’s welfare within the constraints of treatment scarcity.^3^ This approach can be applied in the context of mAb therapeutics to determine allocation policies, using patient factors such as age, race and ethnicity, comorbidities, COVID-19 vaccination status, as well as interactions between these factors to achieve allocations that minimize hospitalization rates across the population. In theory, for future public health emergencies, electronic health record data platforms could be linked to ongoing PL, enabling allocation policies to be updated flexibly during a period of scarcity to address emerging conditions that were not present in initial trials. During the COVID-19 pandemic, these included vaccine introduction, emergence of new variants, and authorization to use scarce mAbs in new populations.

We aimed to develop a point-allocation scoring system that uses PL to allocate limited mAb treatment capacity, minimizing the overall hospitalization rate across a population and improving health equity. Additionally, we sought to compare the PL approach with other allocation methods used during the pandemic to determine the best allocation approach that could be adapted and implemented for therapeutic shortages in the future.

## Methods

### Study Design and Population

This retrospective cohort study leveraged observational cohorts that represented a collaboration between the University of Colorado researchers, University of Colorado Health leaders, and the Colorado Department of Public Health and Environment. We used a data platform, consisting of electronic health record (EHR) data merged with statewide vaccination and mortality data.^4,5,6^ The Colorado Multiple Institutional Review Board approved this study with a waiver of informed consent because the data were deidentified. This study adheres to Strengthening the Reporting of Observational Studies in Epidemiology (STROBE) reporting guideline. Data were analyzed between from January 2023 to May 2024.

### Training and Testing Cohorts

The training cohort included all patients with a positive SARS-CoV-2 test with documented mAb FDA EUA–qualifying conditions derived from EHR data between October 1, 2021, and December 11, 2021. This period occurred during the largest rise of the Delta variant phase of COVID-19, when Colorado implemented crisis standards of care due to health care system strain and limited resources.^7^ We removed individuals who were already admitted to the hospital at the time of their positive SARS-CoV-2 test result. Additionally, we included patients meeting identical criteria between June 1, 2021, and October 1, 2021, as a testing cohort to assess the generalizability of the developed allocation point system under treatment scarcity. The predominant variant in Colorado during these periods was Delta (B.1.617.2). Notably, between June and October 2021, mAb shortages were severe within the health care system, but between October and December 2021, administration capacity of mAbs increased. Detailed clinical justification for choosing these 2 data periods as the training cohort vs testing cohort is in eAppendix 1 in Supplement 1. We also conducted a sensitivity analysis of covariate importance and point-system distribution to compare the data-splitting approach that we used to a random-splitting method.

### Clinically Important Covariates

We included clinically important variables available during the treatment and allocation decision-making period for predicting 28-day hospitalization, including age, sex, race and ethnicity, insurance status, obesity status, immunosuppressed status, hypertension, pulmonary disease, kidney disease, cardiovascular disease, diabetes, and vaccination status.^4,5,6^ For building the allocation scheme, we accounted for race, ethnicity, and insurance as potential confounders in the causal treatment effect model. However, we excluded these variables when constructing policy learning trees (PLTs) to reduce the potential legal ramifications of using race and ethnicity or insurance in a policy allocation.^8^

### PLT-Based Allocation System

PL aims to find the treatment allocation rule that, if implemented, would optimize a predefined outcome across a set of allocation rules.^3^ We identified an allocation rule that minimizes overall hospitalization. We used PLTs as a specific PL model.

### PL and PLTs

We first fit a causal forest, a random forest algorithm that estimates causal risk differences conditioning on patient covariates (eFigure 1 and eAppendix 1 in Supplement 1).^3^ We then used PLTs to learn the optimal allocation policy over the training cohort by modeling the treatment effect (causal risk difference) modification by covariates (eFigure 1 in Supplement 1; stage 2). We trained a PLT ensemble using resampling and out-of-bag samples to reduce variability and overfitting.^9^ In the testing cohort, treatment allocation decisions used majority voting across the PLT ensemble (eAppendix 1 in Supplement 1).

### Covariate Importance

To understand the roles of covariates in PLT allocation, that is, which and how patient covariates modify patients’ responses to treatments, we summarized frequencies of covariates (excluding race, ethnicity, and insurance) during tree splitting among trees in PLTs ensemble. This covariate modification information guided the development of the point systems used in this study.

### PLT-Based Allocation Points

**Figure 1.**
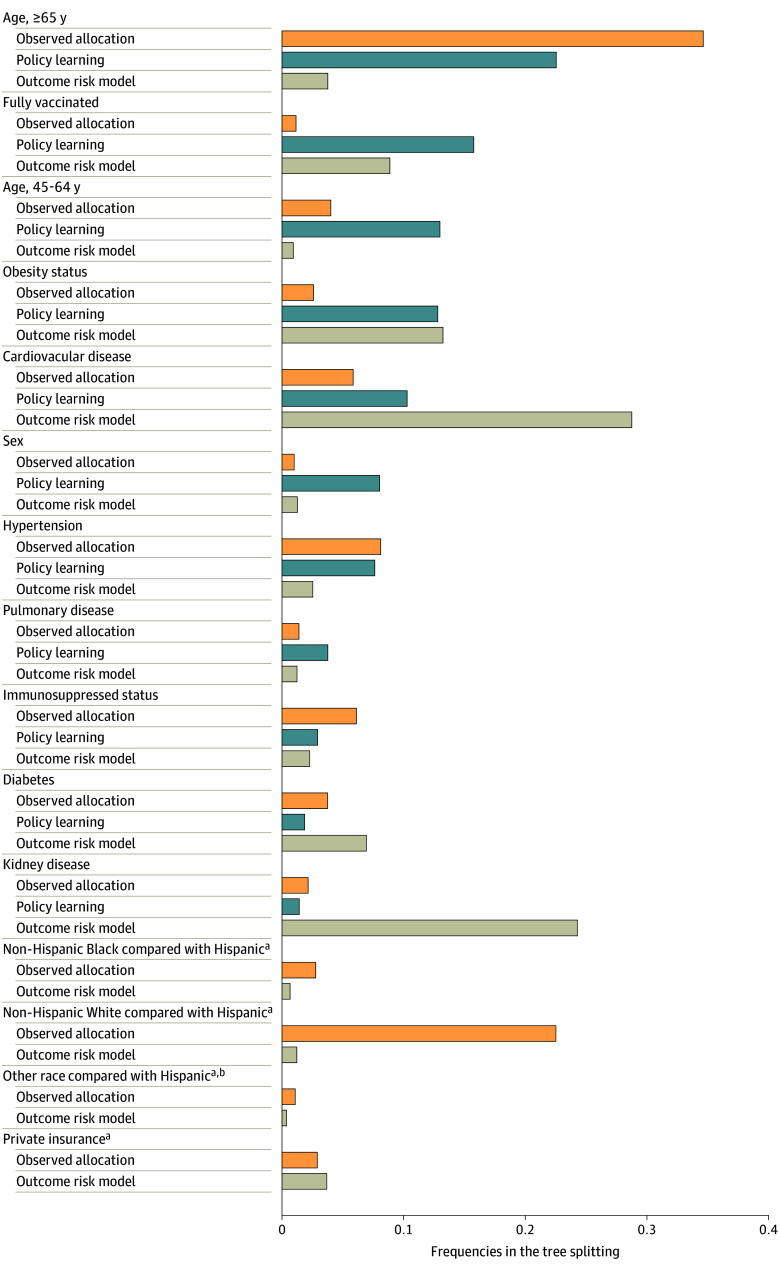
Covariate Importance During Policy Learning Frequencies in tree splitting were determined with the weighted sum of frequencies of a covariate split at each depth in the policy learning tree ensemble. ^a^Policy learning trees excluded these variables during allocation. ^b^The other category included patients who reported their race to be American Indian or Alaska Native, Asian Indian, Chinese, Filipino, Japanese, Korean, Native Hawaiian and other Pacific Islander, multiracial, other, and those who reported their ethnicity to be non-Hispanic or unknown ethnicity.

To build PLTs into an interpretable clinical point system, we followed a common point system framework that derives points from regression coefficients.^10^ Specifically, we used the nonparametric PLT results in a PLT-based regression model that fits into the existing point system framework. To develop a PLT-based regression model, we used a forward model selection process using the top 5 important variables in PLTs (Figure 1; aged 65 years or older; fully vaccinated; aged 45 to 64 years; obesity; and cardiovascular disease) and patient covariate interactions from the best PLT (eFigure 4 in Supplement 1). For this, we used a metric in the PL literature to identify (eAppendix 1 in Supplement 1).^3^ Then, we converted PLT-based regression coefficients into points proportional to mAb treatment effects (eAppendix 1 in Supplement 1).^10^

**Figure 2.**
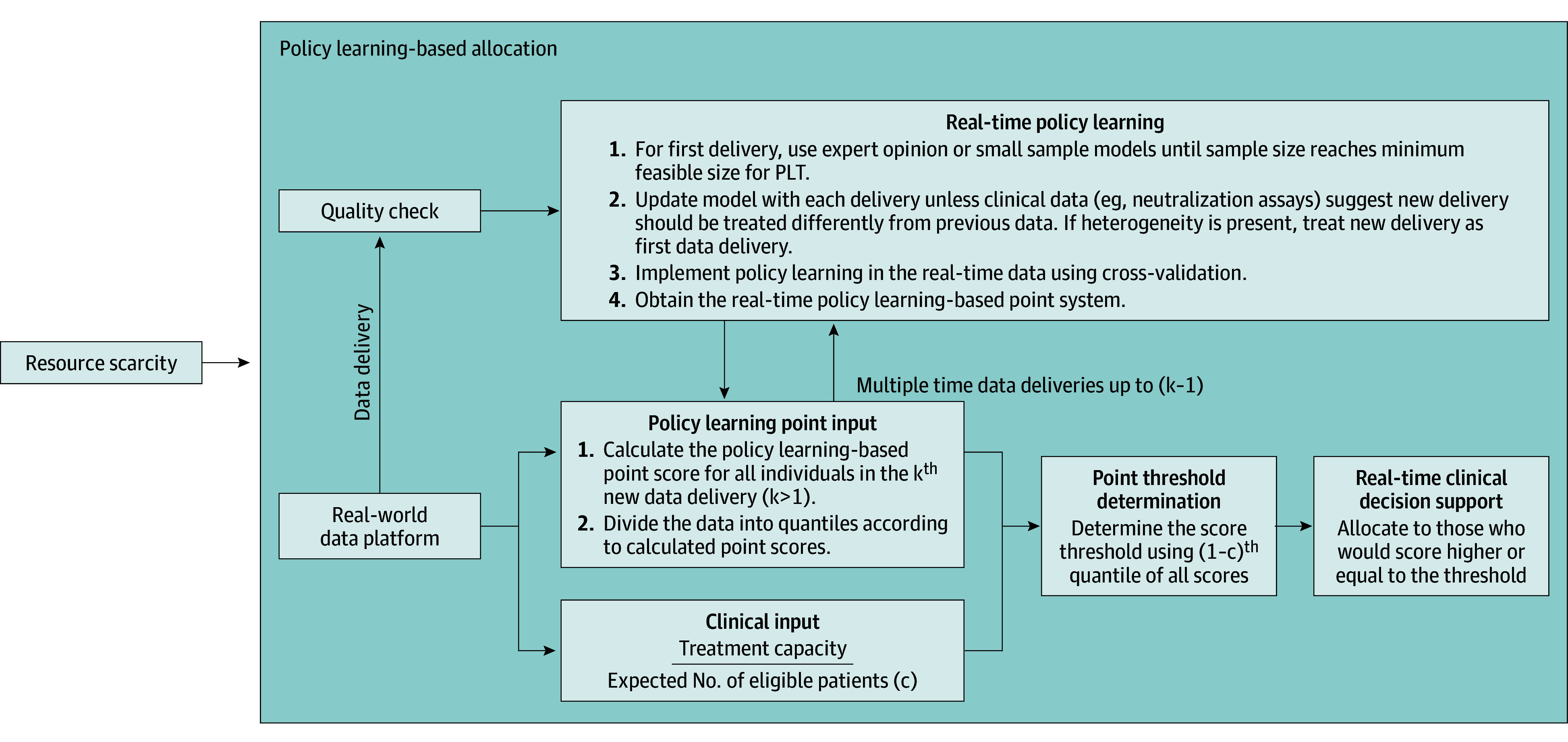
Allocation System Based on Policy Learning Tree (PLT) Assignment and Score Under Resource Constraints Treatment capacity may reflect an estimate of the total number of treatments available or the rate-limiting factor in providing treatment (eg, staffed infusion chairs per week).

Next, we illustrated how to use this point system with real-time clinical inputs, such as treatment capacity and expected number of eligible patients (Figure 2). We first calculated an allocation score for each patient in the testing data cohort based on the point system. Then, the allocation system determined the allocation score threshold according to a clinical input value. Options for the allocation threshold included an estimate of the proportion of treatment allocation among the total number of patients who were seeking care, considering factors such as treatment availability and the expected number of affected populations. We let such a proportion be *c* and calculated the allocation score threshold according to the *(1 − c)^th^* all allocation scores among individuals in the testing data cohort. We compared the PLT-based point system used in this study to the Monoclonal Antibody Screening Score (MASS) used by the Mayo Clinic for identifying eligible individuals for mAb. MASS stratified individuals by hospitalization risk by assigning points to each original FDA EUA criterion from November 2020, as previously described.^2^ The maximum score is 18 with higher scores indicating greater hospitalization risk.

### Performance Metrics

We used the reduction in hospitalization as the primary performance measure. To evaluate PLTs vs observed allocation, we calculated causal risk difference and causal number needed to treat (NNT) to avert hospitalization within 28 days of diagnosis. We visually compared the risk reduction of hospitalization if a point system had been used at a range of treatment proportions compared to no treatment over the entire testing cohort (eAppendix 1 in Supplement 1).

## Results

### Study Population

#### Training Cohort

Of 15 372 nonhospitalized patients with a positive SARS-CoV-2 test result from October 1, 2021, to December 10, 2021, a total of 9542 met the study inclusion criteria. Among the 9542 in the training cohort, 5418 patients (56.8%) were female, 4151 (43.5%) were aged 18 to 44 years, 3146 (33.0%) were aged 45 to 64 years, and 2245 (23.5%) were 65 years or older. Of the 9542 patients eligible for mAb treatment, 3862 patients (40.5%) received mAbs, and 5680 (59.5%) did not receive mAbs (eFigure 2 in Supplement 1). Patients treated with mAbs generally reflected the characteristics of patients at high risk for progression to severe COVID-19 in this cohort (Table 1). The individuals treated with mAbs, compared to the individuals who were not treated with mAbs, had higher proportions of individuals who were 65 years or older (1304 [33.8%] vs 941 [16.6%]), were non-Hispanic White (2983 [77.2%] vs 3468 [61.1%]), had Medicare insurance (1310 [33.9%] vs 953 [16.8%]), and had 2 or more comorbid conditions (1329 [34.4%] vs 1300 [22.9%]). Although some covariates had different distributions between the treated and untreated groups, the inverse probability weighting in the PL method accounts for these confounding effects (eFigure 1 in Supplement 1, stage 1).^3^

**Table 1.** Characteristics of the Training Cohort^a^

Characteristic	No. (%)		
	mAb treated	mAb untreated	Total
Patients, No.	3862	5680	9542
Age group, y			
18-44	1153 (29.9)	2998 (52.8)	4151 (43.5)
45-64	1405 (36.4)	1741 (30.7)	3146 (33.0)
≥65	1304 (33.8)	941 (16.6)	2245 (23.5)
Sex			
Female	2192 (56.8)	3226 (56.8)	5418 (56.8)
Male	1670 (43.2)	2454 (43.2)	4124 (43.2)
Race and ethnicity			
Hispanic	542 (14.0)	1372 (24.2)	1914 (20.1)
Non-Hispanic Black	147 (3.8)	432 (7.6)	579 (6.1)
Non-Hispanic White	2983 (77.2)	3468 (61.1)	6451 (67.6)
Other^b^	190 (4.9)	408 (7.2)	598 (6.3)
Insurance			
Private/commercial	2053 (53.2)	3408 (60.0)	5461 (57.2)
Medicare	1310 (33.9)	953 (16.8)	2263 (23.7)
Medicaid	322 (8.3)	974 (17.1)	1296 (13.6)
Uninsured	96 (2.5)	145 (2.6)	241 (2.5)
Other/unknown	81 (2.1)	200 (3.5)	281 (2.9)
Immunosuppressed			
Mild	470 (12.2)	506 (8.9)	976 (10.2)
Moderate/severe	477 (12.4)	370 (6.5)	847 (8.9)
Obesity	1200 (31.1)	1431 (25.2)	2631 (27.6)
No. of other comorbid conditions			
1	1222 (31.6)	1861 (32.8)	3083 (32.3)
≥2	1329 (34.4)	1300 (22.9)	2629 (27.6)
Diabetes	662 (17.1)	647 (11.4)	1309 (13.7)
Cardiovascular disease	723 (18.7)	648 (11.4)	1371 (14.4)
Pulmonary disease	1219 (31.6)	1602 (28.2)	2821 (29.6)
Kidney disease	352 (9.1)	322 (5.7)	674 (7.1)
Hypertension	1582 (41.0)	1739 (30.6)	3321 (34.8)
Liver disease			
Mild	320 (8.3)	353 (6.2)	673 (7.1)
Severe	38 (1.0)	32 (0.6)	70 (0.7)
No. of vaccinations before SARS-CoV-2 positive test result			
0	1936 (50.1)	3027 (53.3)	4963 (52.0)
1	243 (6.3)	387 (6.8)	630 (6.6)
2	1369 (35.4)	2048 (36.1)	3417 (35.8)
≥3	314 (8.1)	218 (3.8)	532 (5.6)
28-d Hospitalizations	142 (3.7)	271 (4.8)	413 (4.3)

Abbreviation: mAb, monoclonal antibody.

^a^ The training cohort consisted of patients with a positive COVID-19 test result from October 1, 2021, and December 11, 2021.

^b^ The other category included patients who reported their race to be American Indian or Alaska Native, Asian Indian, Chinese, Filipino, Japanese, Korean, Native Hawaiian and other Pacific Islander, multiracial, other, and those who reported their ethnicity to be non-Hispanic or unknown ethnicity.

#### Testing Cohort

A total of 6248 eligible patients in the testing cohort had a positive test result for SARS-CoV-2 from June 1, 2021, to September 30, 2021. In this cohort, 3416 (54.7%) were female, 2827 (45.2%) were aged 18 to 44 years, 1927 (30.8%) were aged 45 to 64 years, and 1494 (23.9%) were 65 years or older (eTable 1 in Supplement 1). Among patients who met the inclusion criteria, 1329 patients (21.3%) received mAb treatment (eFigure 3 in Supplement 1). Demographic and clinical characteristics of the testing cohort were similar to those of the training cohort. Sensitivity analysis showed that results were robust to a random training and testing splitting approach (eFigure 5 and eAppendix 2 in Supplement 1).

### Reduction of Hospitalization From PLTs

Treatment allocation under the PLTs ensemble led to an estimated 1.6% reduction (95% CI, −2.0% to −1.2%) in overall expected hospitalization compared to observed treatment allocation in the testing cohort (NNT, 63 individuals [95% CI, 41 to 120 individuals]). The expected overall hospitalization rate was estimated to be 6.0% (95% CI, 5.0% to 7.1%). A top-performing PLT led to the greatest expected reduction in hospitalizations when allocating mAb to patients who were not fully vaccinated, had cardiovascular disease, were not immunosuppressed, and had obesity (eFigure 4 in Supplement 1; RD, −0.2% [95% CI, −0.4% to −0.03%]; NNT, 5 individuals [95% CI, 3 to 30 individuals]).

### Patient Covariate Importance During PL

Figure 1 shows that both the observed and PLT allocation methods identified being 65 years or older as a potentially highly influential factor in reducing hospitalization with treatment allocation. Being fully vaccinated, between 45 and 65 years of age, and with obesity were found by PLT-based treatment allocation to potentially influence how treatment reduces hospitalization; however, these factors were not identified by the observed treatment allocation. In clinical practice, treatment allocation tends to go to non-Hispanic White individuals with hypertension or immunocompromised status. Race and ethnicity were excluded from consideration in PLT-based allocation. However, in a sensitivity analysis that included race and ethnicity variables in PLT-based allocation, the top 5 important variables for predicting treatment effect remained the same (eFigure 5 in Supplement 1).

The PLTs can solve a fundamental flaw of standard scoring approaches that use outcome risk models: the lack of treatment information (eFigure 1 in Supplement 1; stage 1). Thus, existing methods that solely rely on outcome risks could overlook patients who benefit most from treatments but do not have a high-risk profile for hospitalizations without treatments.^2^ For example, individuals 65 years or older are not at high risk for hospitalization but would observe a large treatment effect on hospitalization (Figure 1). In situations of resource scarcity, effective treatment allocation involves understanding how the treatment reduces the burdens on the health care system for various patient subgroups. This understanding forms the basis of the PLT-based allocation system.

### Reduction of Hospitalization by the PLT-Based Allocation Point System

**Figure 3.**
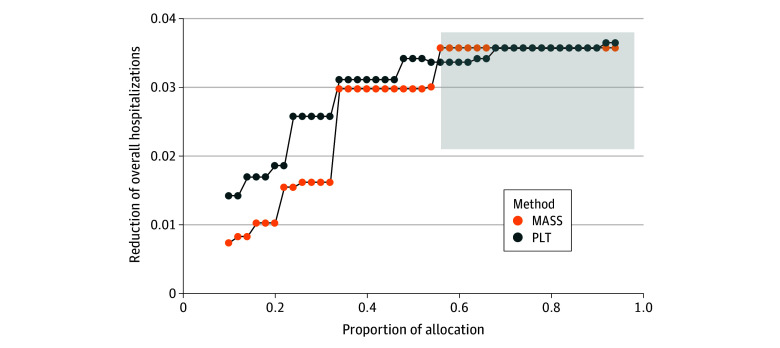
Improvement in 28-Day Hospitalization Comparing Allocation Systems The comparisons of 2 point systems were under different proportions of monoclonal antibody treatment allocation among 6428 eligible patients in the testing cohort. Monoclonal Antibody Screening Score (MASS) was developed at Mayo Clinic to quickly identify and stratify patients by hospitalization risk by assigning points to each original emergency use authorization criterion from November 2020.^2^ The testing cohort included patients with a positive SARS-CoV-2 test result from June 1, 2021, to September 20, 2021. In the gray region, MASS allocates treatments to everyone, and policy learning tree (PLT)–based point systems still have scoring thresholds based on the proportion of allocation.

Table 2 shows the final PLT-based point system developed from the final model in eTable 2 in Supplement 1. Compared to a baseline hospitalization rate where no one received treatment in the testing cohort (6.7% [95% CI, 6.2% to 7.2%]), the PLT-based point system reduced 28-day hospitalization more than MASS (maximum overall hospitalization difference, −1.0% [95% CI, −1.3% to −0.7%]) across various treatment thresholds (Figure 3). The PLT-based point system observed a larger benefit than MASS when mAbs are scarcer (eg, when less than 35% of eligible patients can receive mAbs). Additionally, MASS allocated treatments to the whole testing cohort after the treatments were available for more than 55% of the population (Figure 3 and eTable 3 in Supplement 1). In contrast, a PLT-based point system still prioritizes treatments for populations under the same scarcity scenario. In Supplement 1, eTable 3 shows that the population PLT-based point system allocates treatments to have a smaller NNT than MASS, suggesting that PLT-based point systems better optimize overall treatment efficiency as measured by the reduction in hospitalization (1/NNT).

**Table 2.** Comparison of Allocation Systems

Variable	Point
**PLT-based point system**	
Age ≥45 y to <65 y	4
Age ≥65 y	5
Cardiovascular disease	5
Obesity	3
Fully vaccinated	−1
Kidney disease	7
Pulmonary	2
Cardiovascular disease × fully vaccinated	−7
Obesity × fully vaccinated	−5
Cardiovascular disease × obesity	5
Age ≥45 y to <65 y × obesity	3
Age ≥45 y to <65 y × fully vaccinated	−3
**MASS** ^a^	
Age ≥65 y	2
Obesity	2
Diabetes	2
Kidney disease	3
Cardiovascular disease × age ≥55 y	2
Pulmonary disease × age ≥55 y	3
Hypertension × age ≥55 y	1
Immunosuppressed	3

Abbreviations: MASS, Monoclonal Antibody Screening Score; PLT, policy learning tree.

^a^ MASS was developed at Mayo Clinic to quickly identify and stratify patients by hospitalization risk through assigning points to each original emergency use authorization criterion from November 2020.^2^

## Discussion

In this retrospective cohort study, we demonstrated that a PLT-based protocol for allocating mAbs has the potential to optimize the population effectiveness of mAb therapy in reducing overall hospitalization during periods of treatment scarcity. Further, we applied a commonly used point system during the period, finding that implementing a point system also would have resulted in reduced hospitalizations compared to the observed data. In comparing usual allocation and another point system approach (MASS), the PLT approach resulted in the lowest rates of hospitalizations, with gains over both the usual care (observed) allocation and the MASS point system. The benefit of PL over observed data amounts to an NNT of 63 patients to prevent 1 hospitalization. These data highlight the potential for implementing real-time data platforms with PL to maximize the impact of allocation on critical population outcomes during scarcity.

Periods of scarce supply of several COVID-19 mAb treatments prompted the implementation of various allocation systems across the US. First-come, first-served approaches, defined by a lack of allocation policy, were common given the ease of implementation; however, such approaches risk exacerbating existing health care disparities and inequities and may not optimize population benefit in periods when hospital inpatient capacity was severely limited.^11,12^ Lotteries, used in some systems, had the advantage of providing equal opportunity to receive mAbs for all eligible patients, which aims to address equity concerns. Still, some have suggested that a weighted lottery (in which patients from disadvantaged areas might have higher odds of being selected) could be used to further mitigate health disparities.^13^ But unless a lottery was weighted for clinical characteristics, it could not account for differences in expected clinical effectiveness (eg, those with lower risk for severe disease would have an equal chance of treatment as someone with very high risk) and, therefore, might produce worse population health outcomes. When clinicians express concerns about allocation, sharing the details of PLTs with them can help with understanding behind-the-scenes models and support clinicians more than an uncertain lottery.^11,14^ Despite clinician concerns about allocation, policymakers and health care systems would decide when to activate and deactivate PLTs with evolving conditions. Although our analysis uses longer time periods, in practice, the PLT would look back over a set period initially and be updated iteratively.

Some health care centers used point systems, allocating mAbs to those reaching a threshold score and assigning points to specific clinical or sociodemographic factors based on regression or univariate analyses.^15^ Such scoring typically accounts for few possible interactions, even though these interactions might be very important. For example, patients with specific combinations of comorbid conditions (eg, obesity and lung disease) might be especially likely to benefit from a treatment that is in short supply, while other combinations involving obesity may be less likely to benefit from treatment. PLT-based learning methods can address these complex interactions that frequently occur in patients. Linkage of a PLT protocol to an EHR platform could provide updated allocation information under evolving conditions, such as the emergence of new variants and variable uptake of vaccination.

The evolving nature of pandemics leaves treatment efficacy unknown and requires ongoing data quality checks and modeling adaptations. Furthermore, several technical considerations are applied to PLTs (Figure 2). The first is the data and model quality checks, such as suitable sample sizes, which simulations may help to determine; additional investigation, such as exploring the use of penalized regression when sample size is small, is warranted (eAppendix 3 in Supplement 1). The second is integrating new data deliveries. One option is to continuously update the model with new data deliveries and use existing information, such as in vitro neutralization assays, to inform changes in data and treatment effectiveness. PLT-based allocation should optimize benefits, and it can use previous data to model a covariate’s modification of patient responses to therapies (eAppendices 1 and 3 in Supplement 1). PLT-based allocation should use the best available data to predict allocations; if not known, then expert opinions are warranted until better predictions can be made. Lastly, point systems should address patient demand, including patient preference and trust. Models can be explained to patients using an individual patient benefit graph (eFigure 7 and eAppendix 2 in Supplement 1) and can be optimized for patient- and clinician-facing materials through community engagement and user interface design. The system can also derive individual patient points for each treatment option in multiarm settings (eAppendix 3 in Supplement 1).

When resource constraints occur, prioritizing treatments according to intervention efficiency is especially important.^16^ The PLT approach that we tested was designed to allocate treatments to optimize overall population treatment benefits according to covariates’ impact on our estimated treatment effect (RD, risk of hospitalization in treated − risk of hospitalization in untreated), rather than only looking at variables associated with the risk of outcomes in general. We found that expert opinion may misalign with the most important variables for predicting hospitalization risk. Immunocompromised status has been frequently used in clinical settings to determine mAb treatment allocation and was among the top 5 variables driving usual care allocation. However, PLTs suggest immunocompromised status was relatively unimportant in PLT-based allocation in preventing overall hospitalization, consistent with previous work from our health system showing minimal benefit.^4,5,17,18,19^ Discrepancies between expert opinion and data-driven allocation highlight the need to implement real-time, data-driven allocation policies.

### Limitations

This study has important limitations for consideration. First, these methods may be subject to confounding by unknown and unobserved confounders. Importantly, we controlled only variables in the EHR, which are likely to be readily available for clinical decision-making. However, the results of this study may not be generalizable to other health systems, and in fact, the methods used herein may optimize allocation only for a given local population. The proposed method could be tailored and implemented on a state or even local health system level. As the PLT-based point system does not include race and ethnicity variables, the results of this study may have limited generalizability for different racial and ethnic distributions. However, allocation strategies based on patient factors other than race and ethnicity performed similarly to models that included race and ethnicity. Given that most of the population in our study consisted of non-Hispanic White adult patients, the PLT-based score system may have allocated differently with a more diverse population, including pregnant patients or pediatric patients. To better address health inequities, future allocation schemes should prioritize including metrics that reflect systematic health and social inequities, such as the Social Vulnerability Index.^20^

Furthermore, the improvements in the reduction of hospitalization by PLTs are likely overestimates, as this model assumes optimal allocation can be achieved in clinical practice. However, such allocation alone is not possible owing to many barriers to providing and receiving treatments, especially among historically marginalized communities. In addition to the technical considerations of running PLTs, the PLT-based point system needs to come with multimodal implementation and dissemination strategies to address the specific needs and barriers faced by marginalized communities. Strategies, such as tailored patient outreach messaging, visual aids, access-to-care barrier reductions, community partnership in the dissemination of treatment evidence, clinician education regarding treatments and allocation, and government support, are necessary to promote the uptake of and equitable access to treatments.^11,21,22,23,24^

## Conclusions

In this retrospective cohort study using data from an EHR platform of nonhospitalized patients with COVID-19, we found that PLT-based allocation resulted in greater risk reductions in hospitalization than other point scoring systems, performing maximally under conditions of greater treatment scarcity. The PLT-based method may overcome biases intrinsic to allocation variable selection by expert opinion or simplistic statistical approaches. A PLT-based method can be flexibly updated as public health emergencies, such as a pandemic, evolve. This research emphasizes the importance of creating robust, real-time data platforms for informed, data-driven resource distribution during periods of therapeutic scarcity.

## Notes

Supplement 1. eFigure 1. Policy Learning Procedure eFigure 2. Flow of Patients Into the Training Data Cohort eFigure 3. Flow of Patients Into the Testing Data Cohort eFigure 4. Policy Learned Allocation Tree Applied to the Training Data eFigure 5. Sensitivity Analysis of Covariate Importance eFigure 6. Sensitivity Analysis of Point Distribution to a Random Data Splitting eFigure 7. Individual Patient Benefit Graph eFigure 8. Policy Learning Tree Performance With Different Training Sample Sizes eAppendix 1. Methods Details eAppendix 2. Additional Results eAppendix 3. Practical Consideration of Real-Time PLT-Based Allocation During Resource Scarcity eTable 1. Patient Characteristics Description in the Testing Cohort eTable 2. Point System Final Regression Model eTable 3. Allocation Thresholds and Number Needed to Treat Among Allocated Population Under Point Systems eReferences

Supplement 2. Data Sharing Statement
