# Supplementary material for: A Machine Learning Method for Allocating Scarce COVID-19 Monoclonal Antibodies
Source: JAMA Health Forum. 2024 Sep 13;5(9):e242884. doi: 10.1001/jamahealthforum.2024.2884 (PMC11400220; doi:10.1001/jamahealthforum.2024.2884)
Supplement: Supplement 1. — eFigure 1. Policy Learning Procedure eFigure 2. Flow of Patients Into the Training Data Cohort eFigure 3. Flow of Patients Into the Testing Data Cohort eFigure 4. Policy Learned Allocation Tree Applied to the Training Data eFigure 5. Sensitivity Analysis of Covariate Importance eFigure 6. Sensitivity Analysis of Point Distribution to a Random Data Splitting eFigure 7. Individual Patient Benefit Graph eFigure 8. Policy Learning Tree Performance With Different Training Sample Sizes eAppendix 1. Methods Details eAppendix 2. Additional Results eAppendix 3. Practical Consideration of Real-Time PLT-Based Allocation During Resource Scarcity eTable 1. Patient Characteristics Description in the Testing Cohort eTable 2. Point System Final Regression Model eTable 3. Allocation Thresholds and Number Needed to Treat Among Allocated Population Under Point Systems eReferences [file jamahealthforum-e242884-s001.pdf]

Supplemental Online Content

Xiao M, Molina KC, Aggarwal NR, et al. A machine learning method for allocating scarce COVID-19 monoclonal antibodies. *JAMA Health Forum.* 2024;5(9):e242884. doi:10.1001/jamahealthforum.2024.2884

eFigure 1. Policy Learning Procedure

eFigure 2. Flow of Patients Into the Training Data Cohort

eFigure 3. Flow of Patients Into the Testing Data Cohort

eFigure 4. Policy Learned Allocation Tree Applied to the Training Data

eFigure 5. Sensitivity Analysis of Covariate Importance

eFigure 6. Sensitivity Analysis of Point Distribution to a Random Data Splitting

eFigure 7. Individual Patient Benefit Graph

eFigure 8. Policy Learning Tree Performance With Different Training Sample Sizes

eAppendix 1. Methods Details

eAppendix 2. Additional Results

eAppendix 3. Practical Consideration of Real-Time PLT-Based Allocation During Resource Scarcity

eTable 1. Patient Characteristics Description in the Testing Cohort

eTable 2. Point System Final Regression Model

eTable 3. Allocation Thresholds and Number Needed to Treat Among Allocated Population Under Point Systems

eReferences

This supplemental material has been provided by the authors to give readers additional information about their work.

© 2024 Xiao M et al. *JAMA Health Forum.*

eFigure 1. Policy learning procedure.

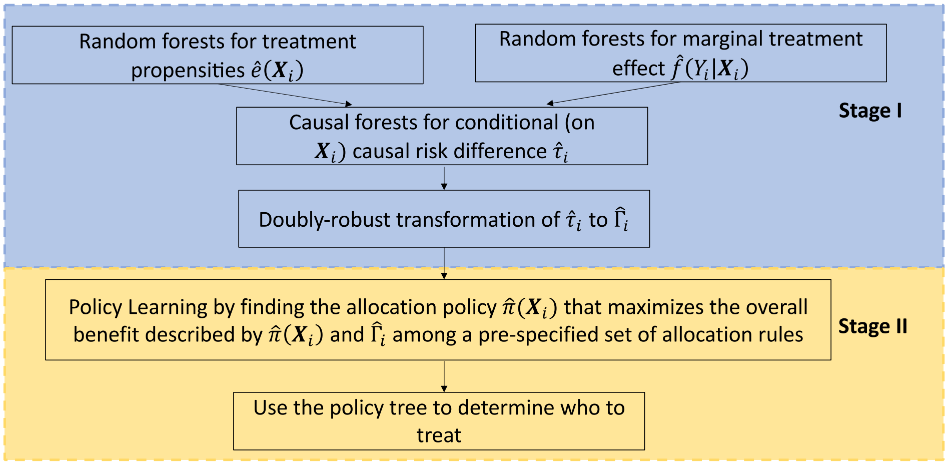

$\boldsymbol{X}_i$ denotes patient covariates for patient i.

© 2024 Xiao M et al. *JAMA Health Forum*.

eFigure 2. Flow of patients into the training data cohort

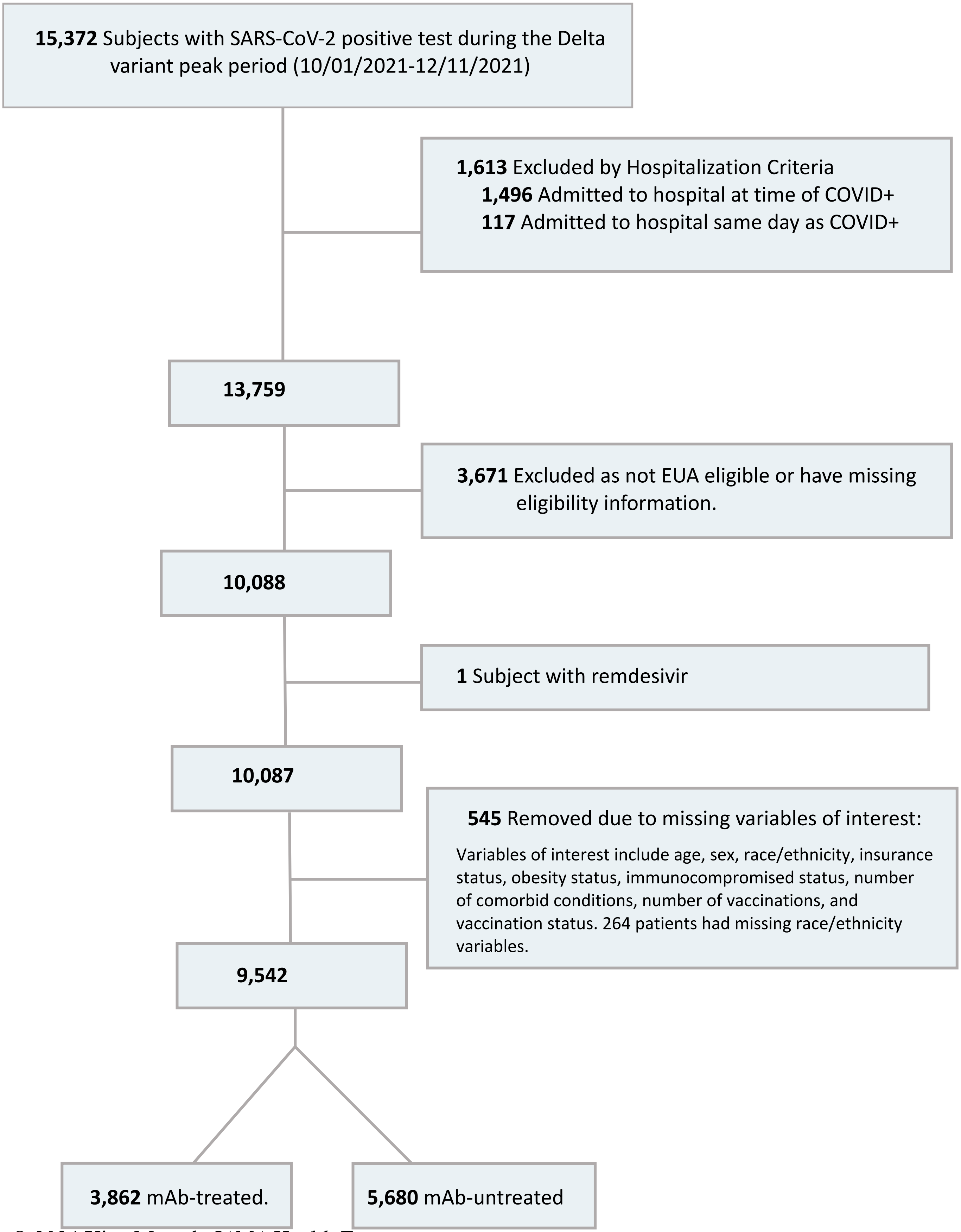

© 2024 Xiao M et al. *JAMA Health Forum.*

eFigure 3. Flow of patients into the testing data cohort

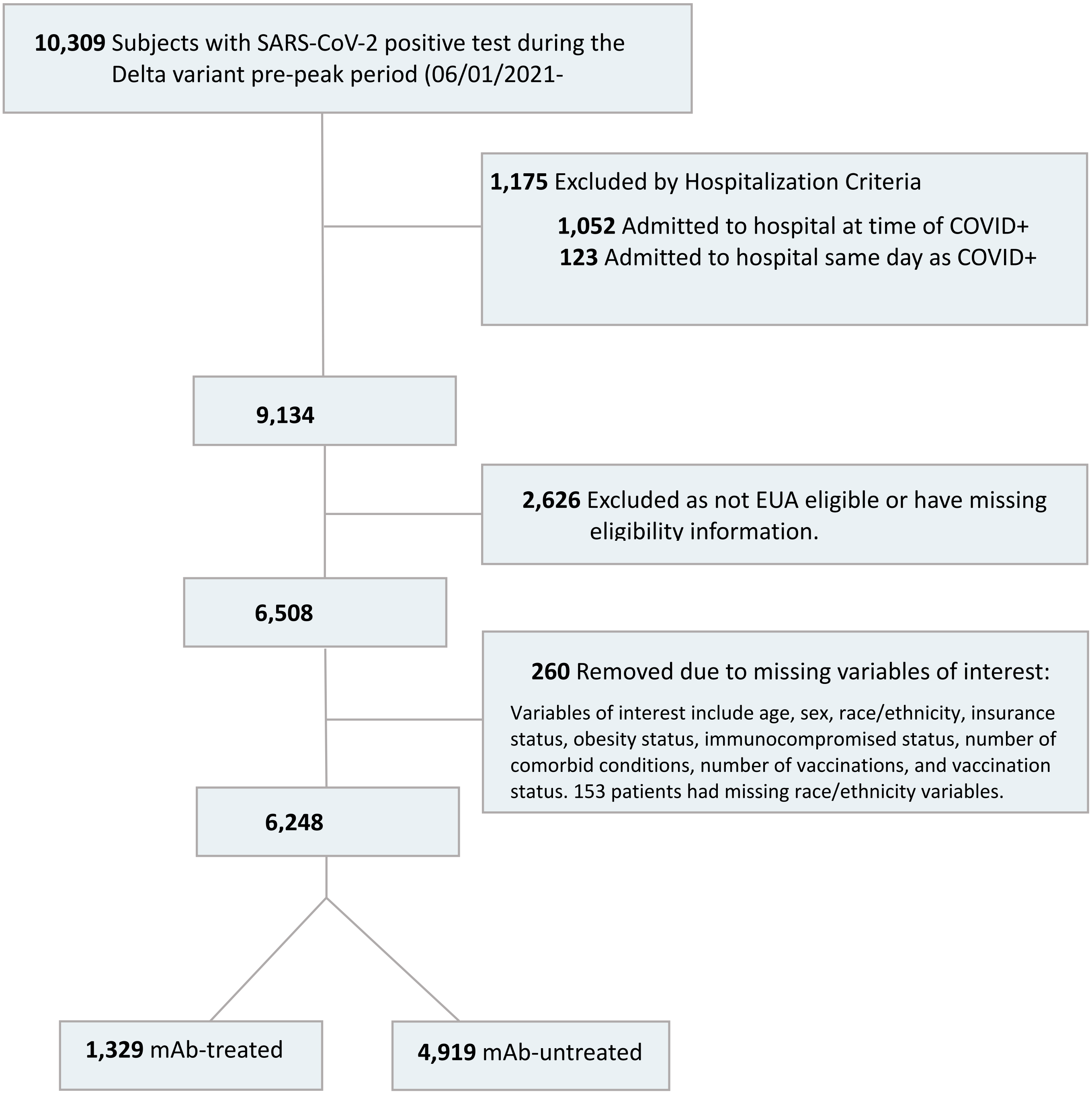

© 2024 Xiao M et al. *JAMA Health Forum.*

eFigure 4. Policy learned allocation tree applied to the training data

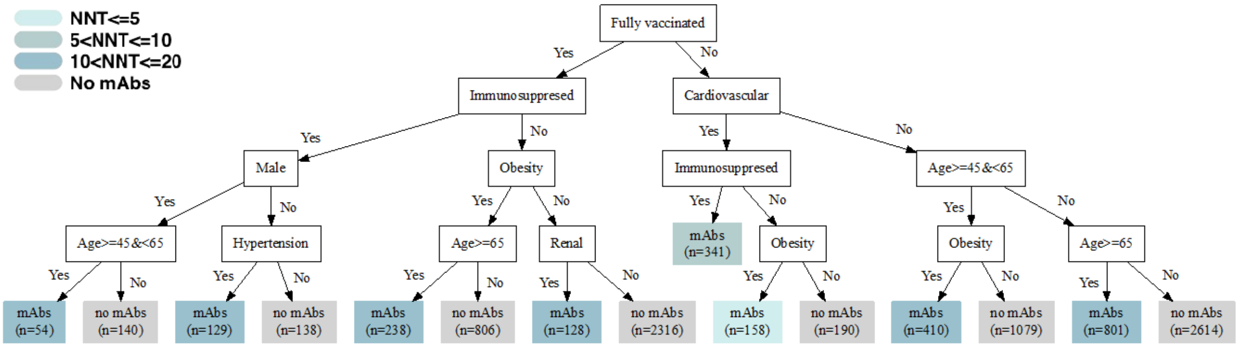

© 2024 Xiao M et al. *JAMA Health Forum.*

eFigure 5. Sensitivity analysis of covariate importance with (A) a random splitting approach to obtain training and testing cohorts and with (B) race variables added into Stage II of Policy Learning. Frequencies in the tree splitting (x-axis): weighted sum of frequencies of a covariate was split on at each depth in the Policy Learning Trees ensemble

(A)

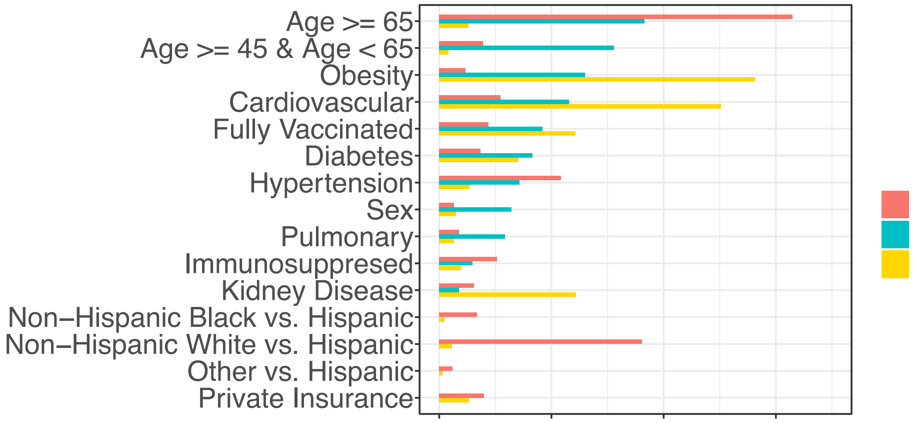

(B)

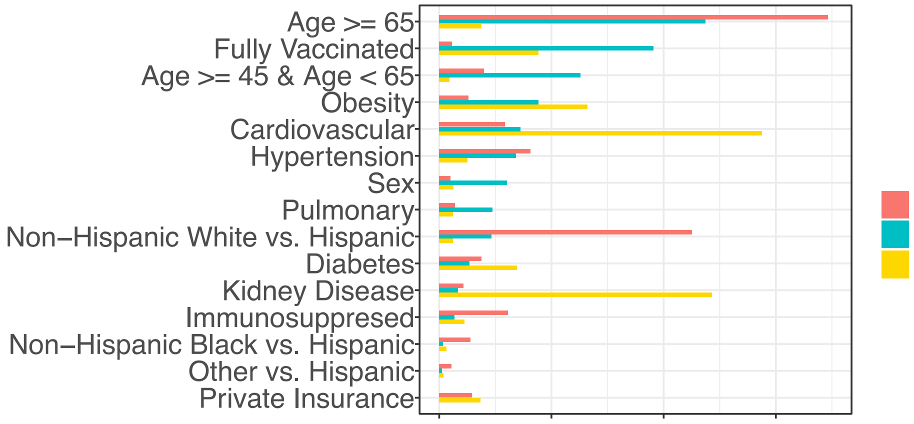

© 2024 Xiao M et al. *JAMA Health Forum.*

eFigure 6. Sensitivity analysis of point distribution to a random data splitting.

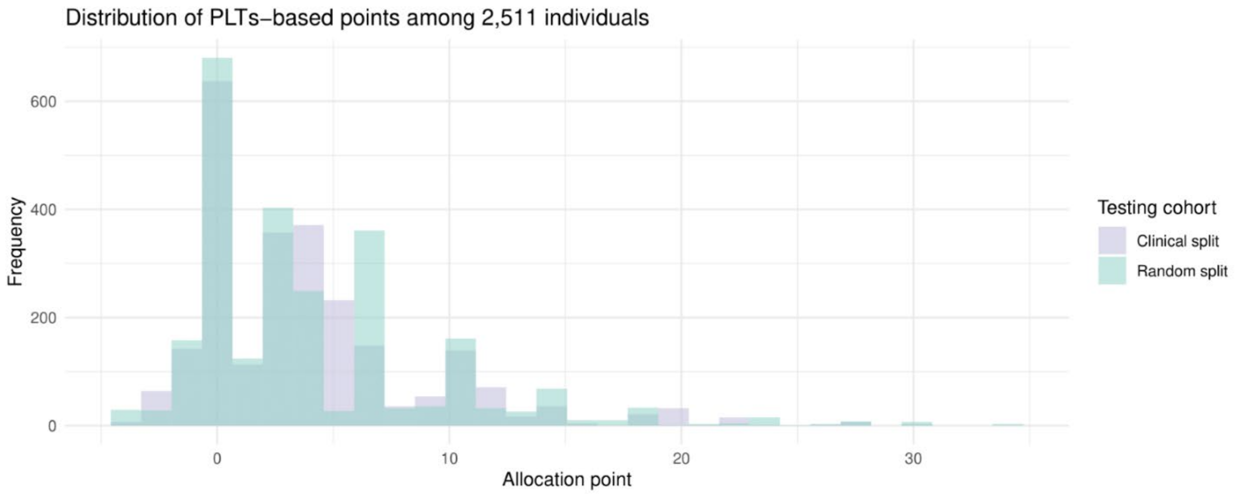

© 2024 Xiao M et al. *JAMA Health Forum.*

eFigure 7. Individual patient benefit graph.

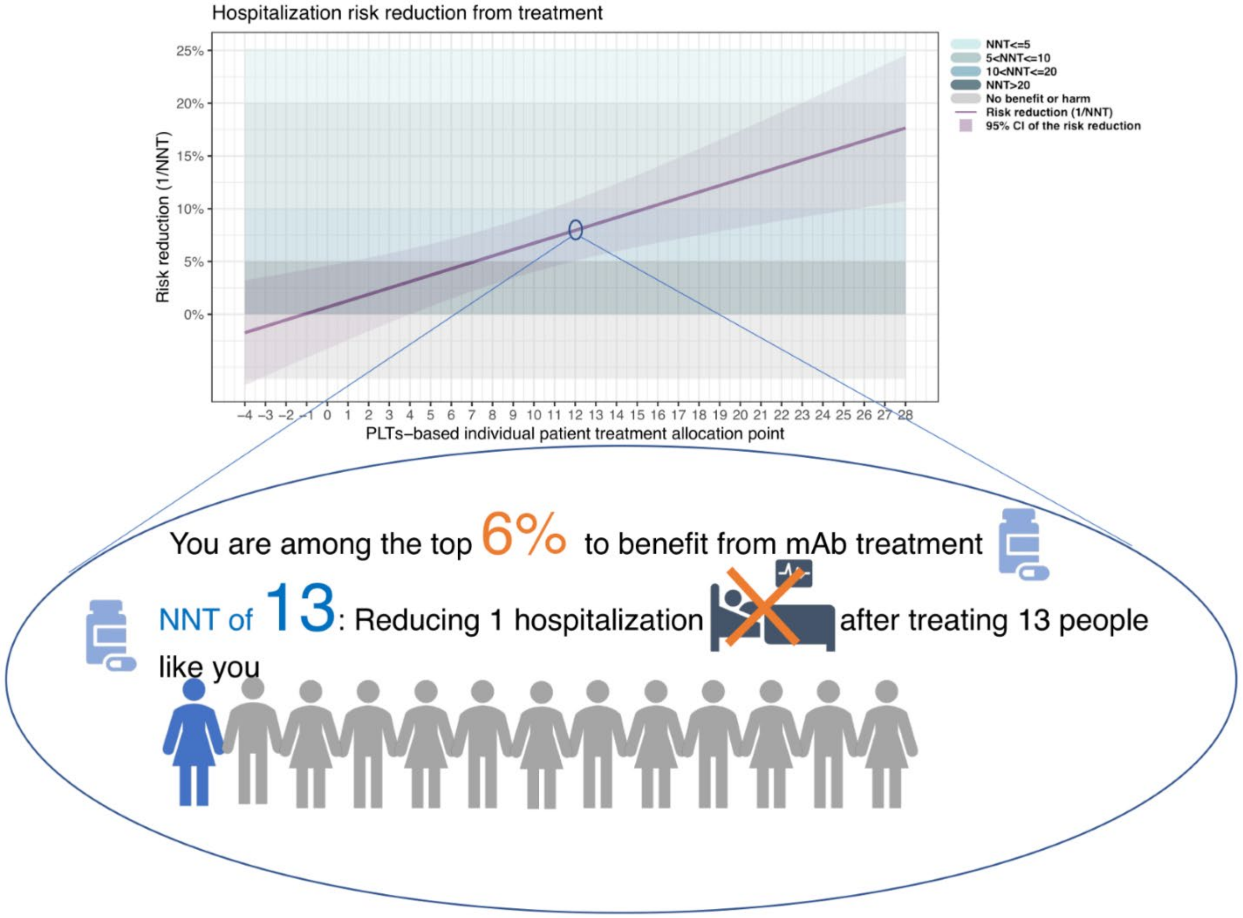

© 2024 Xiao M et al. *JAMA Health Forum.*

eFigure 8. Policy Learning Tree performance with different training sample sizes. The blue dashed line is the optimal risk reduction/policy value if the true and best policy is applied in the testing data.

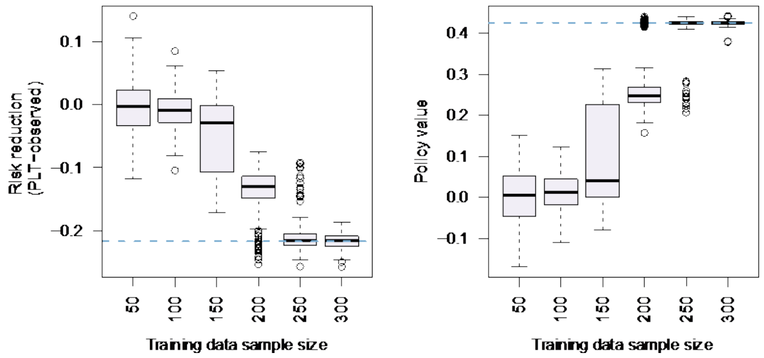

© 2024 Xiao M et al. *JAMA Health Forum.*

eAppendix 1. Methods Details

We first described our training/testing data choice. Then, we outlined details of Policy Learning and Policy Learning Trees, their assumptions, strengths, and limitations. We further elaborated on our innovations, including details behind Policy Learning Trees ensemble (under *Policy Learning Trees*), details behind point systems and its basis on Policy Learning ensemble's covariate importance, and model check details. We also explained methods we used to create the individual patient benefit graph.

A. Training and Testing Data Splitting

We divided our data from 6/2021 to 12/2021 with Delta variant phase of COVID-19 into 10/1/21-12/11/2021 (training) and 6/1/2021-9/30/2021 (testing) according to clinical knowledge about these two time periods and how they fit into the use as the training vs. testing cohort. October to December was the time when Colorado implemented crisis standards of care due to healthcare strain and faced an increased amount of hospitalizations and patient visits than in the summer of 2021.[1] Thus, the sample size of 10/1-12/11 was large (n=9,542), making this period suitable as a training cohort. In addition, 41% of the population were treated among the training cohort (3,912 treated patients) such that PLTs have a large number of treated patients to model the relationship between treatment assignment and patient covariates. We chose June to October period to be the testing cohort because June to October period experienced the most severe mAb shortage, which enabled us to evaluate the proposed method under resource scarcity setting (21% were treated compared to 41% were treated in the training cohort). We still conducted a sensitivity analysis of our model to a random splitting approach and results are in **eAppendix 2.**.

© 2024 Xiao M et al. *JAMA Health Forum.*

B. Policy Learning

Suppose the patient outcome of interest is Y_i, $W_i \in \{0, 1\}$ is the treatment status, $\boldsymbol{X}_i$ is the vector of patient covariates used for allocation, for each patient i and $i \in \{1, 2, \ldots, n\}$. Policy Learning (PL) models the relationship between observed covariates and the treatment allocation and finds an allocation policy $\hat{\pi}$ among a class of policies Π (e.g., a finite depth of decision trees) that maps patient covariates $\boldsymbol{X}_i^a$ ($\boldsymbol{X}_i^a$ may be a subset of $\boldsymbol{X}_i$) to treatment allocations $\in \{0, 1\}$ which maximizes overall outcomes through a policy value function:[2]

$$\hat{\pi} = argmax_{\pi} \left\{ \frac{1}{n} \sum_i 2(\pi(\boldsymbol{X}_i^a) - 1)\widehat{\Gamma}_i \, : \, \pi \in \Pi \right\}. \tag{S1}$$

$\widehat{\Gamma}_i$ is the estimated doubly robust conditional average treatment effect (DR-CATE) and we would call this value as the causal risk difference for patient i, and $\pi(\boldsymbol{X}_i^a)$ is the allocation based on the policy π using patient covariate values and $\pi(\boldsymbol{X}_i^a) \in \{0, 1\}$ where 0 refers to no treatment and 1 refers to treatment. PL authors[2,3] mentioned that maximizing policy value function is equivalent to optimizing overall outcome and they showed that the maximizer policy $\hat{\pi}$ has a bounded and decayed regret scaled with sample size n. The regret is defined by the difference between the expected outcome by using $\hat{\pi}$ and the best outcome that could be achieved among the policies in the set of allocation rules Π. The policy value function leveraged estimated CATE to find the optimal policy $\hat{\pi}$.

Assumptions. 1) The conditional treatment effect Γ_i is a linear function of average individual counterfactual response. This can be achieved through the doubly robust estimator in Equation S2 (*Policy Learning Trees Implementation Details* below) if the

© 2024 Xiao M et al. *JAMA Health Forum.*

estimates of treatment propensity and outcome model converge sufficiently fast (at the rate of $\frac{1}{\sqrt[4]{n}}$ achieved by most machine learning estimators);[4,5] 2) estimate of counterfactual response and DR-CATE had finite variance and converge to the true value in probability at every possible combination of patient covariates and treatment status; 3) The set of allocation rules Π has a finite dimension (an intuitive analogy is a finite number of parameters needed to specify each policy in Π); 4) The coefficient of $\widehat{m}$ in the linear function to calculate Γ_i is bounded for every combination of patient covariates and treatment status; and Assumptions 1, 2, 3, and 4 behind Stage I below. The primary purpose of our paper is to introduce the PLTs framework for allocating a general treatment; the PLTs model we discussed assumes all mAbs share the same outcome and we combined all mAbs into a general treatment arm.

Strengths. Compared to allocation models that typically model outcome risk factors without treatment information, PL models the effect modification by covariates that inform which covariates an allocation should prioritize and use to achieve the best overall outcome/hospitalizations.[2,3] PL also has theory guarantees for finding a policy with bounded regret, i.e., the difference of policy values between the PL-learned policy and the optimal policy in a pre-specified set of allocation rules. It also applies to settings with unmeasured confounding and instrumental variables.[2] The pre-specified set of allocation rules in PL can be a class of constrained policies such as the k-depth decision trees that we focus on in this paper, outcome constraints, or covariates constraints. Lastly, the PL model can be generalized to multi-arm settings. [6,7]

Limitations. Since PL is a new method, software implementations of PL are currently limited to the class of policy with decision trees in the ‘policytree’ R package. This

© 2024 Xiao M et al. *JAMA Health Forum.*

package requires covariates data to be complete and excludes individuals with missing covariates used for allocation, and this handling of missing data is similar to many prediction models. If the missing mechanism is missing at random, one may use multiple imputation by chained equations to impute the missing data.[8] In addition, it is possible to construct a set of allocation rules with certain constraints manually, but the implementation is not built into existing R packages or other software platforms. Another limitation of PL with a decision tree is that the estimate can have large variations with small changes in data, which motivated us to develop a Policy Learning Tree ensemble to address this problem.

C. Policy Learning Trees

To reduce the variability of policy learning results, we built an ensemble of 130 policy learning trees (PLTs) and each policy is presented as a decision tree. The PLT-based allocation was obtained from the majority voting and was assessed by the expected overall hospitalization reduction from treatment in the testing cohort. To determine the best-performing policy tree structure that maximizes overall treatment effect under allocation, we evaluated each of the policy trees in the corresponding out-of-bag (OOB) samples,[9] where the performance metric is the policy value function $\frac{1}{n_{OOB}}\left\{\sum_i 2(\hat{\pi}(\boldsymbol{X}_i^a) - 1)\widehat{\Gamma}_i\right\}$ from Equation S1. OOB samples represented 36%-38% of the training data among those 130 rounds of re-sampling.

D. Policy Learning Trees Implementation Details

Steps to obtain $\hat{\pi}$ in each of the 130 PLTs are:

© 2024 Xiao M et al. *JAMA Health Forum.*

i. Observed allocation and outcome model (Stage I): Get estimated treatment propensity $\hat{e}(\boldsymbol{X}_i)$ and estimated marginal outcome $\hat{f}(Y_i|\boldsymbol{X}_i)$ through random forests for $W \sim X$ (observed allocation model) and $Y \sim X$ (outcome model) among the bagged training samples.

ii. Causal forest model (Stage I): Define $\widetilde{Y}_i = Y_i - \hat{f}^{-i}(Y_i|\boldsymbol{X}_i)$ and $\widetilde{W}_i = W_i - \hat{e}^{-i}(\boldsymbol{X}_i)$ where the $\hat{f}^{-i}(Y_i|\boldsymbol{X}_i)$ and $\hat{e}^{-i}(\boldsymbol{X}_i)$ come from trees in random forests where patient i is in the out-of-bag sample set. Then, obtain CATE $\hat{\tau}_i$ by minimizing the loss function $argmin_{\tau}\left\{\frac{1}{n}\sum\left(\widetilde{Y}_i - \widetilde{W}_i\tau(X_i)\right)^2 + \lambda_n(\tau)\right\}$ where $\lambda_n()$ is the regularization term for the complexity of $\tau(\cdot)$ function. This step is done through causal forest. Each tree in the causal forest used about 50% of the training data to build a tree where child nodes were developed by maximizing treatment effect differences between nodes. The tree used sub-samples (50% of the data in building a tree) from the samples for treatment effect estimation to determine whether to split into child nodes. It uses an adaptive weight $a_i(\boldsymbol{X}_i)$ in a Robinson estimator to estimate CATE τ_i,[3,10] where $a_i(\boldsymbol{X}_i)$ captures the frequency of training samples fall into the same leaf as patient with covariates $\boldsymbol{X}_i$.

iii. Doubly-robust estimator (Stage I): Use a doubly robust estimator of the conditional treatment effect (DR-CATE) of $\widehat{\Gamma}_i$ by augmented inverse probability weighting among OOB samples:[2]

$$\widehat{\Gamma}_i = \hat{\mu}(\boldsymbol{X}_i, 1) - \hat{\mu}(\boldsymbol{X}_i, 0) + \frac{W_i - \hat{e}(\boldsymbol{X}_i)}{\hat{e}(\boldsymbol{X}_i)\left(1-\hat{e}(\boldsymbol{X}_i)\right)}\left(Y_i - \hat{\mu}(\boldsymbol{X}_i, W_i)\right), \quad \text{(S2)}$$

$$\hat{\mu}(\boldsymbol{X}_i, 1) = \hat{f}(Y_i|\boldsymbol{X}_i) + \left(1 - \hat{e}(\boldsymbol{X}_i)\right)\hat{\tau}_i,\ \hat{\mu}(\boldsymbol{X}_i, 0) = \hat{f}(Y_i|\boldsymbol{X}_i) - \hat{e}(\boldsymbol{X}_i)\hat{\tau}_i,$$

© 2024 Xiao M et al. *JAMA Health Forum.*

$$\hat{e}(\boldsymbol{X}_i) = \hat{g}(W_i = 1|\boldsymbol{X}_i),$$

where all functions were estimated from the bagging samples (non-OOB samples) and $\hat{\mu}(\boldsymbol{X}_i, \cdot)$ is the estimate of counterfactual response under treated (1) or untreated (0).

iv. Policy Learning Tree (Stage II): Pre-specify the set of allocation rules Π then find $\hat{\pi}$ by Equation S1. We pre-specified the policy tree class to a classification/decision tree with depth 4 and an exact tree search depth of 3 and minimal leaf node size of 50 samples. Then, we used policy tree search algorithm that search a decision tree policy $\hat{\pi}$ that maximizes $\frac{1}{n_{OOB}}\left\{\sum_i 2(\hat{\pi}(\boldsymbol{X}_i^a) - 1)\widehat{\Gamma_i}\right\}$.

Steps i-iii are Stage I and step iv is Stage II in **eFigure 1** where steps i-ii and steps iii-iv used different data. Steps i-ii apply to the re-sampled training data (62%-64% of the training data) and step iii applies to the out-of-bag samples (36%-36% of the training data). In causal forests, we used leave-one-out cross-validation to tune the fraction of samples, number of variables tried at each split, minimum number of observations in each tree leaf, and maximum imbalance of a split in the three random forest models in Stage I, and there were 4000 trees within all forest models. We implemented all Stage I models through the 'grf' R package.[11] During policy learning, we calculated doubly robust treatment effect estimates from causal forest outputs.[12] We then applied the treatment effect estimates as our optimization target to obtain the best allocation policy over depth of 4 decision trees through the 'policytree' R package (**eFigure 1**; Stage II).[7]

© 2024 Xiao M et al. *JAMA Health Forum.*

Assumptions behind Stage I: 1) Stable Unit Treatment Values Assumption. The potential outcomes (e.g., expected hospitalization) of an individual only depend on the individual's received treatment, i.e., $Y_i = Y_i(W_i)$; 2) Treatment is independent with potential outcomes after conditioning on $\boldsymbol{X}_i$ or there is no unmeasured confounding; 3) Positivity assumption, i.e., the treatment propensity is bounded away from 0 and 1; 4) Patient data are independent and identically distributed. The previous subsection discussed detailed assumptions behind Stage II.

Strengths of causal forests. The framework behind Steps i-iii applies to continuous, binary, count, or any outcome type that has a finite second moment.[5] It can estimate CATE in data with missing covariates. It has underlying theory supports for being a consistent estimate of CATE through the doubly robust estimator.[3,5] The framework is flexible in estimating treatment propensities and marginal outcome models, as long as the estimate converges sufficiently fast.[4,5] Thus, it allows us to model interactions and non-linear effects of patient covariates on the observed treatment allocation and the outcome. Here, we focus on using the causal forest which is a powerful tool to estimate conditional average treatment effect in biomedical applications.[13,14] The implementation of causal forests is available in the 'grf' R package.[11]

Strengths of PLTs. Our innovation of the PLTs ensemble from the original PLT in Athey and Wager[2] allows us to analyze important patient covariates and construct a point system after PL procedure, and it uses re-sampling and OOB samples to reduce variability and overfitting compared to a single PLT.[15]

Limitations. A PLT may require a large sample size to achieve the optimal policy value. The estimation of CATE from causal forests also requires a large sample size,

© 2024 Xiao M et al. *JAMA Health Forum.*

especially in the setting of a sparse binary outcome. Tree ensemble methods also can be time-consuming for continuous covariates. The computational time increases with sample sizes, number of covariates, number of levels in covariates, and tree parameters such as the tree depth. Running each tree in a PLT ensemble took about 1.2 minutes. Despite the computational time, we recommend using a validation dataset (e.g., a subsample of OOB samples) to grid search the best parameters for PLT ensemble. Causal forests may not be able to uncover high-dimensional covariate interaction and may violate the positivity assumption with high-dimensional covariates. More extensive studies of the limitations of causal forests are in previous papers.[16,17]

E. Covariate Importance Calculation

We calculated the variable importance as the weighted sum of frequencies of a covariate was split on at each depth among all trees in the PLTs ensemble. Higher weights were given to parent nodes than to child nodes. Specifically, we gave a weight of 1/ depth^2 to node/nodes at each depth of the tree, following the variable importance calculation in the generalized random forest.[11]

F. Policy Learning Trees Point System Details

We developed data-driven point systems based on PLT-based regression models. Specifically, we implemented a forward model selection from a baseline model using the top five important covariates found by PLTs and set the upper bound for the model selection to be a complex regression model suggested by the PLTs ensemble (such as the interaction between the fully vaccinated status and cardiovascular comorbidity). The complex regression model regressed estimated conditional causal risk differences on

© 2024 Xiao M et al. *JAMA Health Forum.*

variables within identified treatment allocation subgroups in the policy tree. Subgroups are defined by variable combinations/interactions that enhance the treatment effect from observed data and lead to treatment allocation in the tree node (see "*Policy Learning"* section). Those variables and the four-/three-/two-way interactions suggested by policy tree are used as the upper complex model for a forward model selection from the baseline model with the top 5 important covariates in PLTs. The model selection threshold uses a p-value of 0.05 to add new variables.[18] Following Sullivan et al., we converted coefficients for variables, including variable interactions, in the final regression models into point systems to predict the causal mAbs treatment effect that guides mAbs allocation.[19] The points given to each variable and the interaction terms were determined by dividing the regression coefficient of each variable by the parameter estimate of the variable with the smallest absolute value and then rounding to the nearest integer.[20,21]

Assumptions. The point system framework of Sullivan et al. depends on a linear regression model between covariate and quantity used for allocation. In our case, we used the doubly robust conditional treatment effect estimate as the allocation principle to model how the covariate modifies the doubly robust conditional treatment effect estimates.

Performance Metric. We compared point systems across various hypothetical treatment proportions to no treatment rather than to the observed allocation, because the observed allocation treated only a specific proportion of the population.

G. PLT-based Allocation for Evolving COVID-19 Conditions

© 2024 Xiao M et al. *JAMA Health Forum.*

The PLTs allocation model focuses on which patient covariates modified the effect of mAb rather than the treatment effect, so it may be less sensitive to the evolving variants and potential lack of mAb utility than the treatment effect analysis. For example, Sotrovimab, which lost its effectiveness during the Omicron period, still had a larger and more significant treatment effect among patients older than 65 years compared to those who were younger than 65 years.[22] This effect modification direction was consistent with the direction of change in mAb treatment effectiveness by age in previous reports.[23]

H. Individual Patient Benefit Graph

We recommend building visualization of PLTs results and infographics that may help to promote equitable access to care, especially in the patient outreach messaging and community partnership in educating the treatment benefits. To visualize PLT's results, we built an individual patient benefit (hospitalization risk reduction) graph where we calculated the causal mean risk reduction from treatment (negative of the causal risk difference) among patients that share the same allocation point in the testing data cohort. We excluded those allocation points if the number of patients that shared the specific allocation point was less than 10 to reduce our results' sensitivity to random noise. Then, we fit a linear regression to regress the mean risk reduction on PLT-based points. We presented these steps here as a simple illustration of converting PLTs into visualization, and refining these steps is still needed in future research.

© 2024 Xiao M et al. *JAMA Health Forum.*

eAppendix 2. Additional Results

A. Data Splitting Sensitivity Analysis Results

We conducted a sensitivity analysis by randomly splitting 60% of the entire data from 6/2021 to 12/2021 (n=15,790) into a training cohort (n=9,474) and the remaining 40% as our testing cohort (n=6,316). The overall expected hospitalization was reduced by 1.4% (95% CI: -2.4%, -0.5%) compared to the observed allocation in the testing cohort after random splitting (the main result showed a reduction of 1.6% with a slightly narrower 95% CI: -2.0%, -1.2%). We also observed that the top important variables in PLTs were similar to our main finding (**eFigure 5A**). Thus, the PLT-based regression model would start from the same base model in the forward model selection process, although the best PLT and its covariate interaction information were different, as expected. Finally, by developing the PLT-based point system from the training data after the random split, we showed the points trained by two data splitting approaches had a similar distribution among 2,511 overlapping testing patients between the random testing cohort and the testing cohort after the clinical split (**eFigure 6**).

B. Interpretation of Individual Patient Benefit Graph

eFigure 7 shows that an increasing hospitalization risk reduction from no treatment (causal risk difference) is associated with higher PLT-based points. Color gradients in the grid corresponded to different levels of number needed to treat (NNT). When incorporating this into patient and provider facing materials through community engagement and user interface design, we suggest explaining NNT and their point's implication from the data we used to build the graph. For example, if a patient had a point score of 12, we found the corresponding risk reduction (benefit). We then inversed

© 2024 Xiao M et al. *JAMA Health Forum.*

this value to get an NNT to be 13 and calculated the proportion of patients who had greater than or equal to this benefit; in this case, 6% of the total patients we have calculated the PLT-based points had a higher or equal benefit from the treatment than this patient. We conveyed the meaning of 6% as “You are among the top 6% to benefit from mAb treatment”, and we explained what their NNT meant by “Reducing 1 hospitalization after treating 13 people like you” with graphics. Since causal risk differences come from doubly robust conditional treatment effects, the interpretation applies to patients who share similar covariates, and we added “people like you” in our NNT interpretation.

© 2024 Xiao M et al. *JAMA Health Forum.*

eAppendix 3. – Practical Consideration of Real-Time PLT-Based Allocation During Resource Scarcity

A. Real-Time Data Quality Checks

Our team has established a real-world data platform that allowed us to produce reliable evidence from bi-weekly electronic health record data deliveries in real-time. Patient-level data was interleaved with statewide vaccination records from the Colorado Comprehensive Immunization Information System, and mortality information from Colorado Vital Records. These data underwent rigorous data quality checks including comparative analysis with previous data deliveries, temporal evaluation of COVID-positive dates in relation to mAb treatment and hospitalization events and imputing where missing, monitoring distribution of mAb treatment types through time and pandemic phase, and comparing the observed data with statewide epidemiological data.

B. Model Quality Checks - Sample Size

The PLT framework here is a nonparametric method that requires a large sample size (though our training and testing samples were both larger than 6000), and it may perform poorly when the training sample size is small. When the training sample size is small, we suggest exploring the use of parametric methods, such as penalized regression, until the sample size reaches a sufficient number determined by simulations.[24,25] For example, we found the sample size of 250 to be sufficiently large for the PLT to reach the optimal performance, through a preliminary simulation (**eFigure 8; Simulation details** below). Users and policymakers can apply this simulation to determine the acceptable amount of difference from the optimal policy/risk reductions (under the best and true policy in the testing data). For example, a training sample size

© 2024 Xiao M et al. *JAMA Health Forum.*

of 250 had near-optimal results via the visual assessment (eFigure 8), and our training sample size (>5,000) was significantly larger than 250, leading to a positive conclusion about our PLT model quality.

Simulation details. We did a preliminary simulation to show that simulations can help to assess feasible sample sizes for a reliable PLT. We designed our simulation parameters to match raw outcome risks we observed in the training cohort, i.e., 3.7% in treated group and 4.8% in untreated group. Data come from the model $Y_i = Y_i(0) + W_i\tau(\boldsymbol{X}_i)$, where we used following distributions to simulation model components: potential outcome for untreated $Y_i(0) \sim Bernouli(1/(1 + e^{-(\frac{1}{20}(X_1+X_2+\cdots+X_7)^2-\frac{1}{10}(X_8+X_9+\cdots+X_{17})+\frac{X_1X_2}{2}+\frac{X_3X_4}{4}+3.55+\epsilon_i)})$, each covariate in $\boldsymbol{X}_i$ is binary and each was simulated from $Bernouli(0.7)$, treatment indicator $W_i \sim Bernouli(1/(1 + e^{-(X_4+X_5+\cdots+X_9-4.2+\epsilon_i)})$, CATE $\tau(\boldsymbol{X}_i) = -\frac{1}{8}(X_1 + X_2 + X_3 + X_4) + X_1X_3 - 0.128$, and random error term $\epsilon_i \sim N(0, 1)$. At a given training sample size (N = 50, 100, 150, 200, 250, 300), we ran 200 simulations to assess whether PLT can produce optimal value function in Equation S1 and optimal hospital risk reduction from the observed outcome risk (the performance metric in the main paper). The sample sizes for the testing data in our evaluation were the same as the training data. We used default parameters of the causal forest function in the 'grf' package and used depth of 4 policy trees. The performance metrics are the policy value function in Equation S1 and the risk reduction from the observed data that we used in the main paper. We followed some of the simulation parameters choices from Athey and Wager[2] and we acknowledge that users should refine our simulation set-ups further.

© 2024 Xiao M et al. *JAMA Health Forum.*

C. Model Quality Checks – Robustness to Sensitivity Analysis

It is important to show conclusion from the model is robust to data variations. We demonstrated the PLT model robustness to adding vs. excluding race variable in the allocation (**eFigure 5**) and to a random splitting of the data (**eAppendix 2. – Additional Results -** *Data Splitting Sensitivity Analysis Results)*.

D. Integrating New Data Deliveries

The second practical consideration is how to integrate new data that may or may not be heterogeneous from existing data. One solution is to update the causal forest model by accumulating data whenever a new data batch reaches a sufficient sample size determined by simulations. The model may evolve over time with changing population and disease characteristics and use preclinical information (e.g., in vitro neutralization assays) to inform the change of data and treatment effectiveness when a new variant arises. If the disease characteristics change or mAb loses utility, we suggest temporarily using previous data deliveries to inform the allocation and switch to new data delivery once it reaches sufficient sample sizes to generate valid results as determined by simulations above. We are asserting that the PLT-based allocation should optimize benefits and it can use previous data to model covariate's modification of patient responses to therapeutics. It should use the best available data to predict allocations – if not known, then an adaptation of PLT is warranted until we can make better predictions.

E. Patient Demand Consideration

Lastly, we acknowledge that the PLT-based point system here focuses on the care resource distribution and places less emphasis on the patient demand in practice, including patient preference and trust. PLT-based point system can be explained to

© 2024 Xiao M et al. *JAMA Health Forum.*

patients using an individual patient benefit graph (**eFigure 7**), as well as working with experts in community engagement and user interface design to optimize patient and provider facing materials. If a patient and clinician face treatment decisions where multiple therapeutics are available but individually are scarce, PLTs can generalize to multi-arm settings and the point system can derive individual patient points for each treatment option.[6,7]

Additionally, simulation studies that thoroughly investigate the proposed PLT framework under various assumptions would be worthwhile for future research. These assumptions include sample size, heterogeneity between new and existing data, resource scarcity, and patient demand.

© 2024 Xiao M et al. *JAMA Health Forum.*

eTable 1. Patient characteristics description in the testing cohort[a]

	mAb Treated (N=1329)	mAb Untreated (N=4919)	Total (N=6248)
**Age Group, n(%)**			
18-44 years	284 (21.4%)	2543 (51.7%)	2827 (45.2%)
45-64 years	457 (34.4%)	1470 (29.9%)	1927 (30.8%)
≥65 years	588 (44.2%)	906 (18.4%)	1494 (23.9%)
**Gender, n(%)**			
Female	708 (53.3%)	2708 (55.1%)	3416 (54.7%)
Male	621 (46.7%)	2211 (44.9%)	2832 (45.3%)
**Race/Ethnicity, n(%)**			
Non-Hispanic White	1087 (81.8%)	3053 (62.1%)	4140 (66.3%)
Hispanic	150 (11.3%)	1084 (22.0%)	1234 (19.8%)
Non-Hispanic Black	31 (2.3%)	423 (8.6%)	454 (7.3%)
Other[b]	61 (4.6%)	359 (7.3%)	420 (6.7%)
**Insurance Status, n(%)**			
Private/Commercial	633 (47.6%)	2847 (57.9%)	3480 (55.7%)
Medicare	564 (42.4%)	902 (18.3%)	1466 (23.5%)
Medicaid	78 (5.9%)	802 (16.3%)	880 (14.1%)
None/Uninsured	30 (2.3%)	187 (3.8%)	217 (3.5%)
Other/Unknown	24 (1.8%)	181 (3.7%)	205 (3.3%)
**Immunosuppressed , n(%)**			
Mild	176 (13.2%)	448 (9.1%)	624 (10.0%)
Moderate/Severe	195 (14.7%)	356 (7.2%)	551 (8.8%)
**Obesity, n(%)**	347 (26.1%)	1167 (23.7%)	1514 (24.2%)
**Number of Other Comorbid Conditions, n(%)**			
One	421 (31.7%)	1583 (32.2%)	2004 (32.1%)
Two or more	524 (39.4%)	1120 (22.8%)	1644 (26.3%)
**Diabetes, n(%)**	250 (18.8%)	575 (11.7%)	825 (13.2%)
**Cardiovascular Disease, n(%)**	273 (20.5%)	586 (11.9%)	859 (13.7%)
**Pulmonary Disease, n(%)**	437 (32.9%)	1273 (25.9%)	1710 (27.4%)
**Renal Disease, n(%)**	171 (12.9%)	278 (5.7%)	449 (7.2%)
**Hypertension, n(%)**	637 (47.9%)	1493 (30.4%)	2130 (34.1%)
**Liver Disease, n(%)**			
Mild	123 (9.3%)	303 (6.2%)	426 (6.8%)
Severe	11 (0.8%)	22 (0.4%)	33 (0.5%)
**Number of vaccinations prior to SARS-CoV-2+ date, n(%)**			
0	671 (50.5%)	3089 (62.8%)	3760 (60.2%)
1	60 (4.5%)	317 (6.4%)	377 (6.0%)
2	561 (42.2%)	1475 (30.0%)	2036 (32.6%)
3+	37 (2.8%)	38 (0.8%)	75 (1.2%)
**28-day hospitalizations, No. (%)**	49 (3.7%)	294 (6.0%)	343 (5.5%)

© 2024 Xiao M et al. *JAMA Health Forum.*

a. The testing cohort was comprised of patients during June 1, 2021, and September 30, 2021.

b. The “Other” category included patients who reported their race as American Indian or Alaska Native, Native Hawaiian and Other Pacific Islander, Asian Indian, Chinese, Filipino, Japanese, Korean, Multiple Race, and Other and who reported their ethnicity as Non-Hispanic or unknown ethnicity.

© 2024 Xiao M et al. *JAMA Health Forum.*

eTable 2. Point system final regression model

Variable name	Coefficient	Standard Error
Intercept	-0.04	0.01
Age ≥ 45 and Age <65	0.04	0.01
Age ≥ 65	0.05	0.01
Cardiovascular	0.05	0.02
Obesity	0.03	0.02
Fully vaccinated	-0.01	0.01
Renal	0.07	0.02
Pulmonary	0.02	0.01
Cardiovascular * Fully vaccinated	-0.08	0.03
Obesity * Fully vaccinated	-0.05	0.02
Cardiovascular * Obesity	0.05	0.03
Age≥45&<65 * Obesity	0.03	0.02
Age≥45&<65 * Fully vaccinated	-0.03	0.02

© 2024 Xiao M et al. *JAMA Health Forum.*

eTable 3. Allocation Thresholds and Number Needed to Treat Among Allocated Population Under Point Systems

**Allocation ratio (c in Figure 1)**	**PLT-based score**		**MASS (Mayo Clinic)**	
	**Point threshold**	**Causal NNT (among treated patients under a specific allocation ratio)**	**Point threshold**	**Causal NNT (among treated patients under a specific allocation ratio)**
0.1	10	12	7	24
0.15	9	12	5	33
0.2	6	17	5	33
0.25	5	17	3	33
0.3	5	17	3	33
0.35	4	22	2	28
0.4	4	22	2	28
0.45	4	22	2	28
0.5	3	23	2	28
0.55	2	30	1	29
0.6	2	30	0	59
0.65	1	32	0	59
0.7	0	50	0	59
0.75	0	50	0	59
0.8	0	50	0	59

*: the difference between the risk of hospitalization that we would have observed if the population had been treated and the risk of hospitalization that we would have observed if the population were not treated. PLT: Policy learning Trees; NNT: Number needed to treat.

© 2024 Xiao M et al. *JAMA Health Forum.*

eReferences

1. Garcia, C. With COVID-19 numbers rising, Colorado activates crisis standards of care. *theweek* https://theweek.com/us/1007053/with-covid-19-numbers-rising-colorado-activates-crisis-standards-of-care (2021).
2. Athey, S. & Wager, S. Policy Learning With Observational Data. *Econometrica* **89**, 133–161 (2021).
3. Wager, S. STATS 361: Causal Inference.
4. Chernozhukov, V. *et al.* Double/debiased machine learning for treatment and structural parameters. *Econom. J.* **21**, C1–C68 (2018).
5. Athey, S., Tibshirani, J. & Wager, S. Generalized random forests. *Ann. Stat.* **47**, 1148–1178 (2019).
6. Zhou, Z., Athey, S. & Wager, S. Offline multi-action policy learning: Generalization and optimization. *Oper. Res.* **71**, 148–183 (2023).
7. Sverdrup, E., Kanodia, A., Zhou, Z., Athey, S. & Wager, S. *Policytree: Policy Learning via Doubly Robust Empirical Welfare Maximization over Trees*. (2022).
8. Azur, M. J., Stuart, E. A., Frangakis, C. & Leaf, P. J. Multiple imputation by chained equations: what is it and how does it work? *Int. J. Methods Psychiatr. Res.* **20**, 40–49 (2011).
9. Hastie, T., Tibshirani, R. & Friedman, J. Random Forests. in *The Elements of Statistical Learning: Data Mining, Inference, and Prediction* (eds. Hastie, T., Tibshirani, R. & Friedman, J.) 587–604 (Springer, New York, NY, 2009). doi:10.1007/978-0-387-84858-7_15.
10. Robinson, P. M. Root-N-Consistent Semiparametric Regression. *Econometrica* **56**, 931–954 (1988).

© 2024 Xiao M et al. *JAMA Health Forum.*

11. Tibshirani, J., Athey, S., Sverdrup, E. & Wager, S. grf: Generalized Random Forests. (2022).
12. Funk, M. J. *et al.* Doubly Robust Estimation of Causal Effects. *Am. J. Epidemiol.* **173**, 761–767 (2011).
13. Raghavan, S. *et al.* Generalizability of heterogeneous treatment effects based on causal forests applied to two randomized clinical trials of intensive glycemic control. *Ann. Epidemiol.* **65**, 101–108 (2022).
14. Broccia, M. *et al.* Heavy prenatal alcohol exposure and overall morbidities: a Danish nationwide cohort study from 1996 to 2018. *Lancet Public Health* **8**, e36–e46 (2023).
15. Hastie, T., Tibshirani, R. & Friedman, J. Random Forests. in *The Elements of Statistical Learning* 587–604 (Springer New York, New York, NY, 2009). doi:10.1007/978-0-387-84858-7_15.
16. Dandl, S. *et al.* What Makes Forest-Based Heterogeneous Treatment Effect Estimators Work? Preprint at http://arxiv.org/abs/2206.10323 (2023).
17. Shiba, K. & Inoue, K. Harnessing causal forests for epidemiologic research: key considerations. *Am. J. Epidemiol.* kwae003 (2024) doi:10.1093/aje/kwae003.
18. Chowdhury, M. Z. I. & Turin, T. C. Variable selection strategies and its importance in clinical prediction modelling. *Fam. Med. Community Health* **8**, 1–7 (2020).
19. Sullivan, L. M., Massaro, J. M. & D’Agostino, R. B. Presentation of multivariate data for clinical use: The Framingham Study risk score functions. *Stat. Med.* **23**, 1631–1660 (2004).

© 2024 Xiao M et al. *JAMA Health Forum.*

20. van Walraven, C., Austin, P. C. & Knoll, G. Predicting potential survival benefit of renal transplantation in patients with chronic kidney disease. *Can. Med. Assoc. J.* **182**, 666–672 (2010).

21. Austin, P. C. & Walraven, C. van. The Mortality Risk Score and the ADG Score: Two Points-Based Scoring Systems for the Johns Hopkins Aggregated Diagnosis Groups to Predict Mortality in a General Adult Population Cohort in Ontario, Canada. *Med. Care* **49**, 940–947 (2011).

22. Aggarwal, N. R. *et al.* Change in effectiveness of sotrovimab for preventing hospitalization and mortality for at-risk COVID-19 outpatients during an Omicron BA.1 and BA.1.1-predominant phase. *Int. J. Infect. Dis.* **128**, 310–317 (2023).

23. Wynia, M. K. *et al.* Real-World Evidence of Neutralizing Monoclonal Antibodies for Preventing Hospitalization and Mortality in COVID-19 Outpatients. *Chest* **163**, 1061–1070 (2023).

24. Imai, K. & Ratkovic, M. Estimating treatment effect heterogeneity in randomized program evaluation. *Ann. Appl. Stat.* **7**, 443–470 (2013).

25. Athey, S. & Imbens, G. Recursive partitioning for heterogeneous causal effects. *Proc. Natl. Acad. Sci.* **113**, 7353–7360 (2016).

© 2024 Xiao M et al. *JAMA Health Forum.*
