# Supplementary material for: A Machine Learning Method for Allocating Scarce COVID-19 Monoclonal Antibodies
Source: JAMA Health Forum. 2024 Sep 13;5(9):e242884. doi: 10.1001/jamahealthforum.2024.2884 (PMC11400220; doi:10.1001/jamahealthforum.2024.2884)
Supplement: Supplement 2. — Data Sharing Statement [file jamahealthforum-e242884-s002.pdf]

Data Sharing Statement

Xiao. A Machine Learning Method for Allocating Scarce COVID-19 Monoclonal Antibodies. *JAMA Health Forum*. Published September 13, 2024. doi:10.1001/jamahealthforum.2024.2884

Data
Data available: Yes
Data types: Deidentified participant data
How to access data: Deidentified participant data and a data dictionary defining each field in the set, will be made available to others with publication upon provision of a signed data access agreement and approval by the project steering committee via communication with the corresponding author.
When available: With publication
